# The Role of the Carnitine/Organic Cation Transporter Novel 2 in the Clinical Outcome of Patients With Locally Advanced Esophageal Carcinoma Treated With Oxaliplatin

**DOI:** 10.3389/fphar.2021.684545

**Published:** 2021-09-16

**Authors:** Dongfeng Sun, Qingfa Chen, Zhibo Gai, Fengxia Zhang, Xiaoqing Yang, Wensi Hu, Chengyu Chen, Guangjie Yang, Severin Hörmann, Gerd. A. Kullak-Ublick, Michele Visentin

**Affiliations:** ^1^ Department of Thoracic Surgery, The First Affiliated Hospital of Shandong First Medical University & Shandong Provincial Qianfoshan Hospital, Shandong Medicine and Health Key Laboratory of Emergency Medicine, Shandong Lung Cancer Institute, Shandong Institute of Respiratory Diseases, Jinan, China; ^2^ The Institute for Tissue Engineering and Regenerative Medicine, Liaocheng University/Liaocheng People’s Hospital, Liaocheng, China; ^3^ Department of Clinical Pharmacology and Toxicology, University Hospital Zurich, University of Zurich, Zurich, Switzerland; ^4^ Department of Pathology, The First Affiliated Hospital of Shandong First Medical University and Shandong Provincial Qianfoshan Hospital, Jinan, China; ^5^ Department of Nuclear Medicine, The Affiliated Hospital of Qingdao University, Qingdao, Shandong, China; ^6^ Department of Pathology and Molecular Pathology, University Hospital Zurich, University of Zurich, Zurich, Switzerland

**Keywords:** biomarker, carnitine transporter, esophageal cancer, oxaliplatin, OCTN2

## Abstract

Esophageal cancer is the ninth most common malignancy worldwide, ranking sixth in mortality. Platinum-based chemotherapy is commonly used for treating locally advanced esophageal cancer, yet it is ineffective in a large portion of patients. There is a need for reliable molecular markers with direct clinical application for a prospective selection of patients who can benefit from chemotherapy and patients in whom toxicity is likely to outweigh the benefit. The cytotoxic activity of platinum derivatives largely depends on the uptake and accumulation into cells, primarily by organic cation transporters (OCTs). The aim of the study was to investigate the impact of OCT expression on the clinical outcome of patients with esophageal cancer treated with oxaliplatin. Twenty patients with esophageal squamous cell carcinoma (SCC) were prospectively enrolled and surgical specimens used for screening OCT expression level by western blotting and/or immunostaining, and for culture of cancer cells. Sixty-seven patients with SCC who received oxaliplatin and for whom follow-up was available were retrospectively assessed for organic cation/carnitine transporter 2 (OCTN2) expression by real time RT-PCR and immunostaining. OCTN2 staining was also performed in 22 esophageal adenocarcinomas. OCTN2 function in patient-derived cancer cells was evaluated by assessing L-carnitine uptake and sensitivity to oxaliplatin. The impact of OCTN2 on oxaliplatin activity was also assessed in HEK293 cells overexpressing OCTN2. OCTN2 expression was higher in tumor than in normal tissues. In patient-derived cancer cells and HEK293 cells, the expression of OCTN2 sensitized to oxaliplatin. Patients treated with oxaliplatin who had high OCTN2 level in the tumor tissue had a reduced risk of recurrence and a longer survival time than those with low expression of OCTN2 in tumor tissue. In conclusion, OCTN2 is expressed in esophageal cancer and it is likely to contribute to the accumulation and cytotoxic activity of oxaliplatin in patients with esophageal carcinoma treated with oxaliplatin.

## Introduction

Esophageal carcinoma is an aggressive malignancy with an overall 5-year survival rate estimated at 15–25% (Pennathur et al., 2013; Siegel et al., 2015; Bray et al., 2018). The two main esophageal carcinoma histopathological subtypes are the squamous cell carcinoma (SCC) and the adenocarcinoma (AC), with the first being predominant in Asia and the second more frequent in Western countries (Zhang et al., 2012; Pennathur et al., 2013). Treatment guidelines of these two histotypes are similar with adjuvant chemotherapy based on platinum derivatives with or without radiotherapy being widely used for the treatment of locally advanced tumors (Herskovic et al., 1992; Cooper et al., 1999). However, although recent data suggest that oxaliplatin is safer, with less sudden death, compared to cisplatin (Huang et al., 2016), the significant increase in treatment-related mortality after surgery remains a limiting factor in platinum-based adjuvant therapy of esophageal cancer (Stahl et al., 2005). Moreover, there are substantial prognostic differences existing according to the response to the therapy, and the current knowledge on the molecular mechanism(s) underlying such variability is still insufficient for a prospective definition of responder and non-responder groups, risk of recurrence and planning of follow-up that is not solely based on histological factors (Vallbohmer and Lenz, 2006; Holscher et al., 2014).

Platinum derivatives in the body are present partly in neutral state and in part as aquated cationic complexes upon replacement of a Cl^−^ with H_2_O (aquation) (Jamieson and Lippard, 1999), thus rendering their cytotoxic activity partly dependent on the expression and function of facilitative transport systems. Indeed, there is extensive experimental evidence, primarily focused on cisplatin, supporting the cardinal role of organic cation transporters in the uptake, accumulation, and cytotoxicity of platinum derivatives. The organic cation transporters 1, 2, and 3 (OCT1-3), encoded by the SLC22A1-3 genes, as well as the organic cation/carnitine transporter 1 and 2 (OCTN1 and N2), encoded by the SLC22A4 and SLC22A5 genes have been shown to transport platinum derivatives *in vitro* (Yonezawa et al., 2006; Filipski et al., 2009; Jong et al., 2011; Koepsell, 2013; Sprowl et al., 2013; Samodelov et al., 2020). Additionally, mice lacking Oct1 and Oct2 treated with cisplatin show no signs of ototoxicity and only mild nephrotoxicity, the most common side effects of cisplatin therapy. Co-medication of wild-type mice with cisplatin and cimetidine, another OCT substrate, protects from cisplatin-induced ototoxicity and partly from nephrotoxicity (Filipski et al., 2009; Ciarimboli et al., 2010). The pivotal role of OCTs in platinum derivatives-induced cytotoxicity is also supported by pharmacogenetic studies. Patients carrying the nonsynonymous single-nucleotide polymorphism (SNP) causing a change from serine to alanine is position 270 of OCT2 amino acid sequence (rs316019), and, in turn, a reduced transport activity, experience milder cisplatin-induced nephrotoxicity (Filipski et al., 2009; Frenzel et al., 2019).

We hypothesized that one or more OCTs might be differentially expressed in esophageal cancer, hence decisive in platinum derivatives accumulation and cytotoxicity. The present work examined the expression level of OCTs in biopsies from patients diagnosed with esophageal cancer and correlated their expression level with the response to oxaliplatin-based chemotherapy.

## Materials and Methods

### Study Approval

The clinical study was conducted according to the Declaration of Helsinki guidelines regarding ethical principles for medical research involving human subjects. All patients provided written informed consent and the study protocol was approved by the Scientific Ethical Committee of The First Affiliated Hospital of Shandong First Medical University, Jinan, China (license number 2013-S068). For the primary culture of SCC cells, the study protocol was approved by the Scientific Ethical Committee of Shandong University, Jinan, China, where the fresh biopsies were obtained (license number SDU2017043). The collection of adenocarcinoma samples was approved by the Ethics committee of the Canton of Zurich, Switzerland (KEK-ZH-2010–0093/0).

### Patients’ Enrollment and Follow-Up

Twenty patients diagnosed with esophageal squamous cell carcinoma (SCC) (age 40–73) and eligible for surgery were prospectively enrolled at The First Affiliated Hospital of Shandong First Medical University between December 2018 and December 2019. Tissues were used for western blotting and/or immunostaining as well as culture of primary cancer cells. Replication of the initial screening findings was performed retrospectively. Sixty-seven samples from patients diagnosed with SCC (age 41–80) admitted to The First Affiliated Hospital of Shandong First Medical University between January 2012 and December 2015, who had been treated with oxaliplatin with available follow-up were assessed for OCTN2 mRNA and protein level, and differential survival analysis. Sixty out of 67 patients underwent partial esophagectomy and regional lymph node dissection; the remainder were not eligible for (presence of metastasis) or refused the surgical treatment. All 67 patients underwent four cycles of oxaliplatin (85 mg/ m^2^, i.v., day 1) combined with 5-fluorouracil (500 mg/ m^2^, i.v., day 1–5) and leucovorin (200 mg/ m^2^, i.v., day 1–5), or gemcitabine (1,000 mg/ m^2^, i.v. weekly for 3 weeks) or docetaxel (75 mg/ m^2^, i.v., day 1). The follow-up workup included physical examination, gastrointestinal barium meal test, blood analysis, CT scans, ultrasonography and gastrointestinal endoscopy. Tumor progression was determined according to clinical and radiological examination. Twenty-one of the patients that relapsed during the follow-up underwent radiotherapy or further chemotherapy that did not include oxaliplatin. Adjacent normal mucosa samples were available for all 87 SCC samples. Twenty-two patients diagnosed with esophageal adenocarcinoma (AC) (age 39–84) were retrospectively identified from the database of the Department of Pathology and Molecular Pathology at the University Hospital of Zurich. For ten patients the adjacent normal esophageal mucosa was available. No follow-up was available for the patients diagnosed with AC. The clinical TNM staging of SCCs and ACs was defined according to the eighth edition of the AJCC/UICC cancer staging manuals (Rice et al., 2017). All studies were observational hence, randomization was not applicable. Relevant clinical information are summarized in Table 1.

**TABLE 1:** Patient characteristics.

Parameters	Total (*n* = 109)	SCC (*n* = 87)	AC (*n* = 22)
Age at diagnosis –yr			
Median (interquartile range)	62 (57–68)	61 (57–67)	68 (63–76)
Range	39–84	40–80	39–84
Sex—no. (%)			
Male	92 (84)	72 (83)	20 (91)
Female	17 (16)	15 (17)	2 (9)
ECOG Performance (%)			
0	59 (54)	59 (68)	0 (0)
1	8 (7)	8 (9)	0 (0)
2	0 (0)	0 (0)	0 (0)
3	0 (0)	0 (0)	0 (0)
4	0 (0)	0 (0)	0 (0)
5	0 (0)	0 (0)	0 (0)
Not reported	42 (39)	20 (23)	22 (100)
Location (%)			
Upper	19 (17)	19 (22)	0 (0)
Medium	32 (29)	32 (37)	0 (0)
Lower	16 (15)	16 (18)	0 (0)
Not reported	42 (39)	20 (23)	22 (100)
Invasion (%)			
T1	12 (11)	8 (9)	4 (18)
T2	22 (20)	17 (20)	5 (23)
T3	64 (59)	52 (60)	12 (55)
T4	11 (10)	10 (11)	1 (4)
Node (%)			
N0	51 (47)	46 (53)	5 (23)
N1	48 (44)	35 (40)	13 (59)
N2	5 (5)	5 (6)	0 (0)
N3	2 (2)	1 (1)	1 (5)
Nx	3 (2)	0 (0)	3 (13)
Metastases (%)			
M0	102 (94)	86 (99)	16 (74)
M1	4 (4)	1 (1)	3 (13)
Mx	3 (2)	0 (0)	3 (13)
Grade (%)			
G1	13 (12)	13 (15)	0 (0)
G2	56 (51)	48 (55)	8 (36)
G3	39 (36)	26 (30)	13 (59)
G4	0 (0)	0 (0)	1 (5)
Gx	1 (1)	0 (0)	1 (5)
Treatment (%)			
Only surgery	20 (18)	20 (23)	0 (0)
Chemotherapy	67 (61)	67 (77)	0 (0)
- Oxaliplatin + Gemcitabine	60	60	0 (0)
- Oxaliplatin + 5-Fluorouracil	4	4	0 (0)
- Oxaliplatin + Docetaxel	3	3	0 (0)
No information	22 (20)	0 (0)	22 (100)
Follow-up (%)			
Available	67 (61)	67 (77)	0 (0)
Not available	42 (39)	20 (23)	22 (100)

### Pathological Assessments and Immunostaining

Tumor tissues and adjacent normal esophageal mucosa were fixed overnight in 10% neutral buffered formalin and then embedded in paraffin. Three-μm sections were stained with hematoxylin and eosin and reviewed independently by two pathologists. Immunostaining was performed on paraffin sections using a microwave-based antigen-retrieval technique. The antibodies against the human OCT1 (LS-C161155, RRID:AB_2889177), OCT2 (LS-C80615, RRID:AB_2191153), OCT3 (LS-C352857, RRID:AB_2889179), and OCTN2 (LS-C681354, RRID:AB_2889180) were purchased from LSBio (Seattle, WA). The anti-OCTN1 (sc-19819, RRID:AB_2191404) was obtained from Santa Cruz Biotechnology (Santa Cruz, CA). Sections were treated with the Envision^+^ DAB kit (Dako, Glostrup, Denmark), according to the manufacturer’s instructions. A semi-quantitative blind method previously described was used to measure the staining intensity and the proportion of positive cells (Sun et al., 2018). Ten visual fields were observed at 400X magnification. One-hundred tumor cells per field were counted. The positive cell scoring systems was based on the percentage of positive cells: <5% (0); 5–25% (1); 25–50% (2); 50–75% (3); >75% (4). The staining intensity was graded as follows: no staining (0); light yellow (1); yellow brown (2); dark brown (3). The sum of the positive cell score and the staining intensity score was further graded as (−), 0–1; (+), 2–3; (++), 4–6; and (+++), 8–12 to obtain the final assessment.

### Total Protein Isolation and Western Blotting

Fifty mg of frozen tissue were homogenized in RIPA buffer (50 mM Tris base, 150 mM NaCl, 1% Nonidet P-40, 0.5% sodium deoxycholate, pH 7.4) supplemented with a protease inhibitor cocktail (Roche Diagnostics, GmbH, Mannheim, Germany). Protein lysates (20 μg) were resolved by SDS-PAGE and blotted onto polyvinylidene difluoride membranes (Millipore, Burlington, MA). The membranes were blocked in 5% non-fat milk/PBS with 0.1% (v/v) Tween 20 (PBS-T), and then incubated overnight at 4°C with the antibody against OCTN2 (LS-C681354, RRID:AB_2889180), followed by 1-h incubation with horseradish peroxidase conjugated goat anti-rabbit IgG (111–035–046, RRID:AB_2337939), purchased from Jackson ImmunoResearch (Cambridgeshire, United Kingdom). β-actin was probed using a mouse monoclonal anti-β-Actin antibody (A2228, RRID:AB_476697, Sigma, St. Louis, MO) and HRP-conjugated Goat Anti-Mouse IgG (ab97023, RRID:AB_10679675, Abcam, Cambridge, United Kingdom).

### Isolation of Total RNA and Relative Quantification by Real Time RT-PCR

Total RNA was isolated from 67 SCC samples and the matched normal mucosa using the TRIzol procedure (Thermo Fisher Scientific, Carlsbad, CA, United States) and quantified at 260 nm. Two micrograms of total RNA were reverse transcribed using oligo (dT) primers and Superscript II retrotranscriptase (Thermo Fisher Scientific, Carlsbad, CA, United States). The cDNA was amplified by real time PCR analysis with the TaqMan universal PCR Master Mix (Thermo Fisher Scientific, Carlsbad, CA, United States) for OCTN2, (Hs00929869_m1, Thermo Fisher Scientific, Carlsbad, CA, United States). Transcript levels, determined from two independent cDNA preparations, were expressed relative to those of the housekeeping gene β-actin (Hs01060665_g1, Thermo Fisher Scientific, Carlsbad, CA, United States).

### Isolation and Culture of Primary Patient-Derived Cancer Cells

Samples from twenty patients who underwent partial esophagectomy were immediately washed in 0.9% NaCl solution, removed, under aseptic conditions, from hemorrhagic necrotic tissue and adipose tissue by 5-min washing in 0.05% chlorhexidine followed by 20-min washing in D-Hank’s solution (containing penicillin 500 units/ ml, streptomycin 500 μg/ ml, amphotericin B 5 μg/ ml). This washing step was repeated 6 times, then the tissue samples were digested in 10% collagenase (Amresco, Solon, OH, United States) in Dulbecco’s modified Eagle’s medium (DMEM) (Thermo Fisher Scientific, Carlsbad, CA, United States). After neutralizing the collagenase with culture medium, cells were spun down for 1 min at 200 g_av_. The cell pellet was seeded in regular DMEM supplemented with 10% fetal bovine serum, 100 units/ ml penicillin, 100 μg/ml streptomycin, cultured at 37°C, 5% CO_2_ changing medium every 48–72 h.

### Culture of Human Embryonic Kidney (HEK293) Cells

Wild-type HEK293 cells were maintained in DMEM supplemented with 10% fetal bovine serum, 100 units/ ml penicillin, 100 μg/ ml streptomycin at 37°C in a humidified atmosphere of 5% CO_2_. HEK-OCTN2 cells were maintained in Geneticin G-418 (600 μg/ ml) as selecting agent (Tamai et al., 2001; Zhang et al., 2020).

### Immunofluorescent Staining of OCTN2 in Primary Patient-Derived Cancer Cells

Cells were seeded onto chamber slides at the density of 10,000 cells/well. When confluent, cells were fixed with 4% formaldehyde in PBS for 15 min at room temperature. Cells were subsequently permeabilized using 0.1% Triton X-100 in PBS for 15 min, and then incubated in blocking solution (PBS containing 0.1% Triton X-100, 3% goat serum, and 2% bovine serum albumin) for 30 min at room temperature, followed by overnight incubation at 4°C with anti-OCTN2 (LS-C681354, RRID:AB_2889180) diluted in PBS containing 0.1% Triton X-100 and 3% goat serum. Cells were washed with PBS, and then incubated for 1 h at room temperature with Alexa Fluor 488 goat anti-rabbit-IgG (A-11008, RRID:AB_143165, Thermo Fisher Scientific, Carlsbad, CA, United States) diluted in PBS-T. Cells were washed and mounted with Crystal Mount (GeneTex, Irvine, CA, United States) for picture acquisition using a Leica DMR upright fluorescence microscope (Leica Microsystems, Wetzler, Germany).

### Uptake Studies in Primary Patient-Derived Cancer Cells

Cells were seeded at the density of 20,000 cells/well onto collagen-coated 6-well plates. Confluent cells were washed and equilibrated in transport buffer (116.4 mM NaCl, 5.3 mM KCl, 1 mM NaH_2_PO_4_, 0.8 mM MgSO_4_, 5.5 mM D-glucose and 20 mM Hepes/Tris, pH 7.4) at 37°C then the buffer was aspirated and transport buffer containing a mixture of non-labeled and labeled L-carnitine (L-[methyl-^3^H]carnitine hydrochloride, specific activity: 64.7 Ci/ mmol, PerkinElmer, China) was added. Uptake was stopped by quick aspiration followed by extensive washing with ice-cold transport buffer. For quantification of the Na^+^-independent uptake, NaCl was replaced with an equimolar concentration of choline chloride (116.4 mM choline chloride, 5. mM KCl, 1 mM KH_2_PO_4_, 0.8 mM MgSO_4_, 5.5 mM D-glucose and 20 mM Hepes/Tris, pH 7.4). Cells were solubilized for 45 min with 1 ml of 1% (w/v) Triton X-100; 500 μl of the lysate was mixed with 4 ml of scintillation liquid (Ultima Gold; PerkinElmer, China) and assessed for intracellular radioactivity by liquid scintillation counting. Protein content was determined by the bicinchoninic acid protein assay.

### Cytotoxicity And Proliferation Assays in Primary Patient-Derived Cancer Cells And HEK293 Cells

Primary patient-derived cancer cells were seeded at the density of 5,000 cells/well onto collagen-coated 96-well plates. After 6 days of culture, cells were treated for 48 h with oxaliplatin at the extracellular concentration of 100 µM. WT- and OCTN2-HEK293 cells were seeded at the density of 10,000 cells/well onto 96-well plates. After 24 h, cells were treated for 48 h with increasing extracellular concentrations of oxaliplatin. Cell viability was monitored by assessing the cellular ATP content by measuring the luminescent signal after lysis with CellTiter-GLO reagent (Promega, Madison, WI, United States). All measurements were performed using a GloMax Discover Microplate Reader (Promega, Madison, WI, United States). For TUNEL staining, esophageal cancer cells were seeded at the density of 10,000 cells/well onto chamber-slides. After 6 days of culture, cells were treated for 24 h with oxaliplatin at the extracellular concentration of 100 μM and then stained using the Click-iT™ TUNEL Alexa Fluor™ 594 Imaging Assay (C10246, Thermo Fisher Scientific, Carlsbad, CA, United States) according to the manufacturer’s protocols. Oxaliplatin-induced changes of intracellular structures were assessed by transmission electron microscope. Esophageal cancer cells were seeded at the density of 20,000 cells/well onto six well plates. After 6 days of culture, cells were resuspended at the density of 50,000/ ml and fixed with 4% glutaraldehyde for 2 h. After washing with PBS, cells were fixed for 1 h in 1% osmium acid solution. Dehydrated with acetone gradient, embedded with 618 epoxy resin, then sections at 70 nm were prepared. After staining with uranium acetate and lead citrate, cells were inspected using a transmission electron microscope (JEM-1200EX, JEOL Ltd., Tokyo, Japan). For cell proliferation studies, primary patient-derived cancer cells were seeded at the density of 10,000 cells/well onto 48 well plate. After 6 days of culture, cells were synchronized for 48 h in serum free medium, then the rate of DNA synthesis was quantified by 5-bromo-2′-deoxyuridine (BrdU) incorporation assay (BD Biosciences, San Diego, CA, United States).

### Statistical Analysis

Shapiro–Wilk test of normality, Chi-square test for independence, univariate and multivariate Cox analyses, and survival curves were calculated using the open access language programming software R (R Project for Statistical Computing, RRID:SCR_001905). The end points evaluated in this study were progression-free survival (PFS) represented by the interval of time from the diagnosis to the recurrence/progression or death at the last follow-up, and overall survival (OS) calculated from the time of the diagnosis to the time of death for any cause or the last follow-up. Patients that did not experience recurrence/progression or death at the end of a 3-years follow-up from initial diagnosis were censored in both PFS and OS analysis. Patients subjected to further therapy after the progression of the disease were censored in OS analysis. Age, tumor location, stage, lymph node status, grade, and OCTN2 protein expression level were tested for association with PFS and OS in univariate analysis. Location, lymph node status and OCTN2 protein expression level were also tested in multivariate analysis. Statistical comparisons of OS and PFS between groups were calculated using the Wald test. Two-tailed unpaired Student t-test and Mann-Whitney test were performed using GraphPad Prism version 8.0 (RRID:SCR_002798). Power analysis was not performed as deemed irrelevant to this study.

## Results

### Expression Level of Organic Cation Transporters in Esophageal Cancer Tissues

**FIGURE 1:**
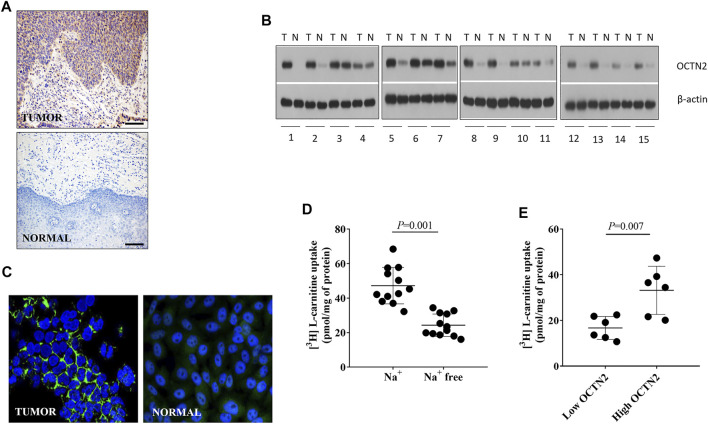
Expression and function of OCTN2 in tumor tissues and in the respective primary cancer cells. Representative immunostaining of tumor tissue and paired normal mucosa. Scale bar = 100 µm **(A)**. Western blot image of total lysates (20 µg) from 15 tumor samples and the respective surrounding normal tissues probed with anti-OCTN2 and anti-β-actin antibodies **(B)**. Representative immunostaining of OCTN2 in primary patient-derived cancer cells and paired normal mucosa. OCTN2 staining is shown in green; nuclei are stained with 4,6-diamidino2-phenylindole (DAPI) in blue. Original magnification, 200 × **(C)**. Five-minute uptake of L-carnitine in primary patient-derived cancer cells in the presence or absence of Na^+^ **(D)**. Na^+^-dependent uptake of L-carnitine in OCTN2 high-expressing and low-expressing patient-derived cancer cells, according to the OCTN2 staining score western blot quantification **(E)**. Each data point represents the uptake values in cells isolated from one tumor tissue and shown as the mean ± S.D. from three independent experiments. Statistical comparison was performed by unpaired Student’s t-test.

OCT expression was screened on an array of tumor tissues obtained from 20 patients diagnosed with SCC who were prospectively enrolled and underwent partial esophagectomy. The expression level of OCT1, OCT2, OCT3, OCTN1, and OCTN2 was assessed by immunohistochemistry in both SCC and adjacent normal tissues. For 15 out of 20 samples, western blotting was also possible. Among them, OCT2, OCT3 and OCTN1 were practically undetectable. There was no obvious difference in the expression level of OCT1 between SCC and the matched normal mucosa (Supplementary Figure S1A; Supplementary Figure S1B). Conversely, OCTN2 was markedly overexpressed in SCC samples as compared with normal mucosa (Figure 1A and Figure 1B).

### Assessment of OCTN2 Function in Primary Patient-Derived Esophageal Cancer Cells

To understand whether OCTN2 detected in the tumor tissues was functioning, cancer cells were successfully isolated and cultured from 12 out of 20 samples. The primary patient-derived cancer cells displayed a positive staining for OCTN2 at the level of the plasma membrane (Figure 1C). Because OCTN2 is a Na^+^-dependent L-carnitine transporter and a Na^+^-independent cation transporter (Samodelov et al., 2020), the function of OCTN2 in primary patient-derived cancer cells was assessed by measuring the uptake of L-carnitine over 5 min in the presence or absence of Na^+^. It can be seen that the intracellular level of L-carnitine was significantly higher when the transport was assessed in the presence of Na^+^, suggesting that OCTN2 in primary cancer cells was active. Noteworthy, the uptake of L-carnitine varied among primary cancer cells isolated from different patients, in line with the varying expression level of OCTN2 measured in the tumor tissues (Figure 1D). When primary cancer cells were grouped according to the expression level of OCTN2 as per western blotting and/or immunostaining (Figure 1A; Figure 1B), the Na^+^-dependent transport of L-carnitine was significantly higher in cells derived from tumor tissues with higher expression of OCTN2 in comparison with that in those isolated from tumors with low expression of OCTN2 (Figure 1E).

### Impact of OCTN2 Expression Level on Cell Proliferation And Oxaliplatin-Induced Cytotoxicity

**FIGURE 2:**
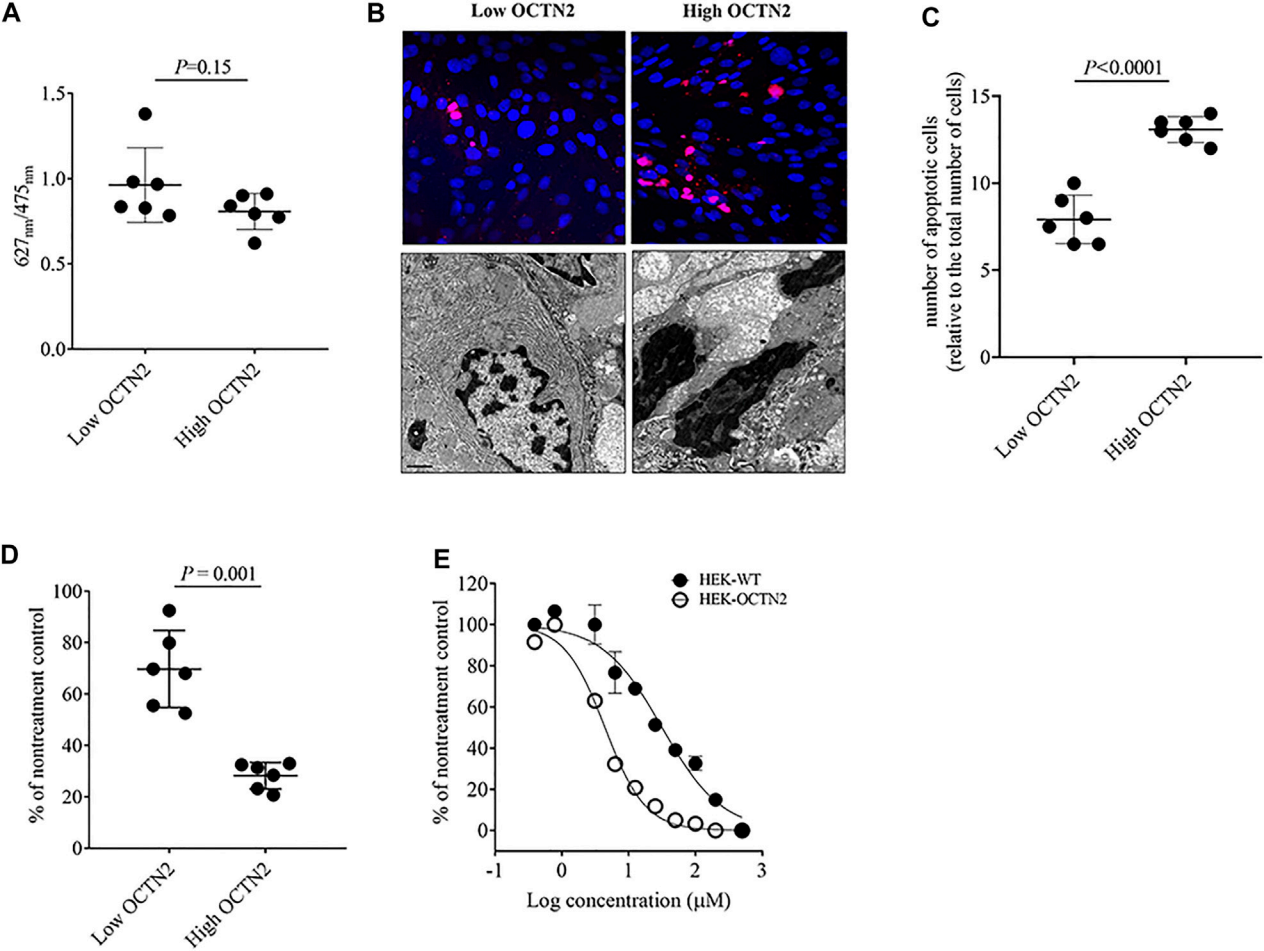
Cell growth and oxaliplatin sensitivity in OCTN2 expressing cells. Cells were grouped according to the tissue staining score and western blot quantification. BrdU incorporation in the DNA of OCTN2 high- and low-expressing cancer cells **(A)**. Representative image of TUNEL staining (**(B)**, top panel) of high- and low-expressing cancer cells after 48-h exposure to oxaliplatin at the extracellular concentration of 100 µM. Representative image electron microscopy (**(B)**, bottom panel) of patient-derived cancer cells after 48-h exposure to oxaliplatin at the extracellular concentration of 100 μM at 10,000x. Quantification of the number of apoptotic cells stained in purple normalized for the number of nuclei, stained with 4,6-diamidino2-phenylindole (DAPI) in blue **(C)**. ATP content in OCTN2 high- and low-expressing patient-derived cancer cells after 48-h exposure to oxaliplatin at the extracellular concentration of 100 µM **(D)**. ATP content in HEK-WT and HEK-OCTN2 exposed for 48 h to increasing extracellular concentrations of oxaliplatin **(E)**. All data are presented as mean ± S.D. from three independent experiments and compared by unpaired Student’s t-test. Curves were modelled using the (log (Inhibitor) vs normalized response-variable slope) equation.

Cancer cell proliferation rate has an established role in tumor growth. Moreover, cells with a high proliferation rate are usually more sensitive to standard cancer chemotherapeutics, including platinum derivatives. To study the impact of OCTN2 on cell proliferation, the incorporation of BrdU in the DNA of primary patient-derived cancer cells was quantified. It can be seen that the proliferation of OCTN2 high- and low-expressing cells was comparable (Figure 2A). Conversely, OCTN2 high-expressing cells appeared to be markedly more sensitive to oxaliplatin, showing an increased number of apoptotic cells as revealed by (i) the higher number of stained cells (Figure 2B top, Figure 2C, (ii) the more evident intracellular structure changes typical of the apoptotic process such as reduced cell volume and nuclear fragmentation (Figure 2B, bottom) and (iii) the reduced ATP content (Figure 2D) as compared with the low-expressing cells. The role of OCTN2 in oxaliplatin-induced toxicity was confirmed in HEK293 cells stably transfected with human OCTN2 (HEK-OCTN2). Expression and function of OCTN2 in the stable transfected clone was previously shown (Tamai et al., 2001; Visentin et al., 2017a; Visentin et al., 2017b). It can be seen that HEK-OCTN2 cells were 6–7 times more sensitive than WT-HEK293 cells (IC_50_ = 4.43 μM, 95% CI 4.02 to 4.87 *vs*. IC_50_ = 29.0 μM, 95% CI 21.9–38.4) (Figure 2E).

### Impact of OCTN2 Expression Level on the Clinical Outcome

**FIGURE 3:**
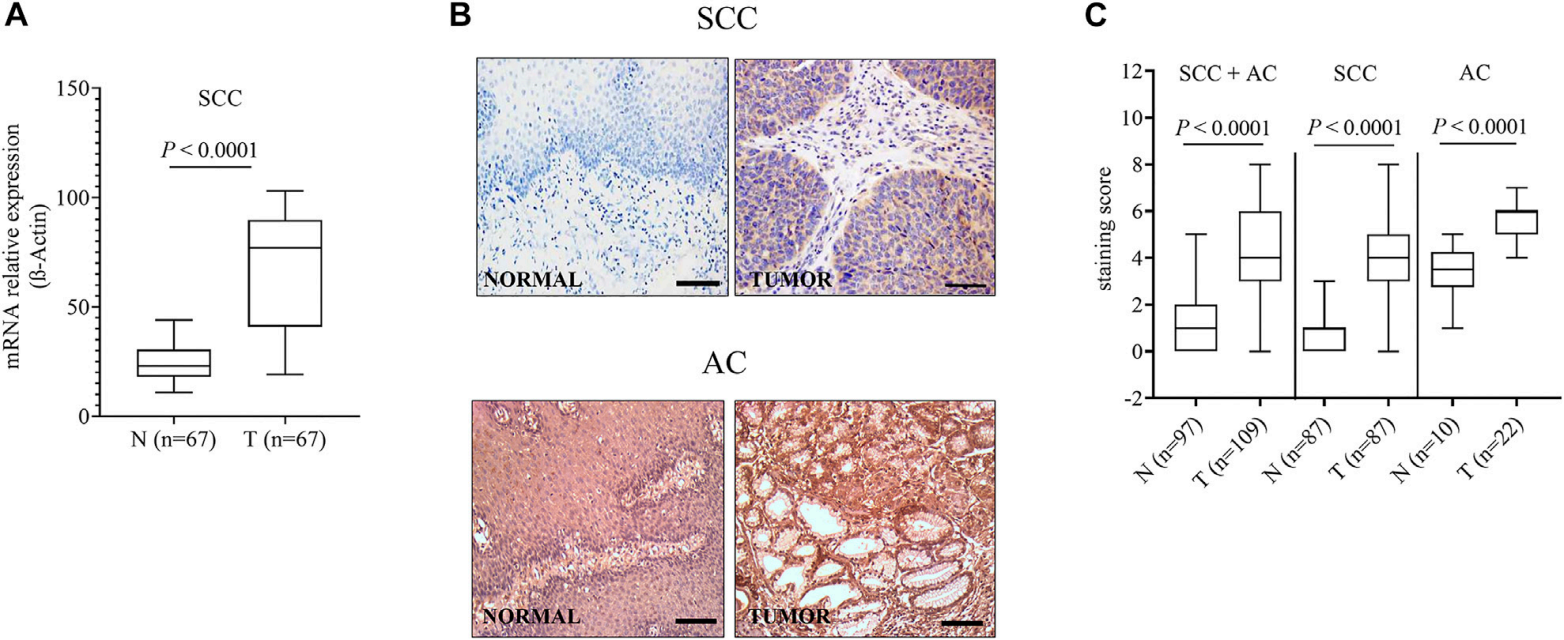
OCTN2 expression level in squamous cell carcinoma (SCC) and adenocarcinoma (AC) samples. OCTN2 mRNA values normalized by the expression of the housekeeping gene b-actin **(A)**. Representative immunostaining of SCC and AC and the respective paired normal tissues. Scale bar = 100 µm **(B)**. Relative quantification of the OCTN2 staining per high-power field. All comparisons were performed by Mann-Whitney test **(C)**.

**FIGURE 4:**
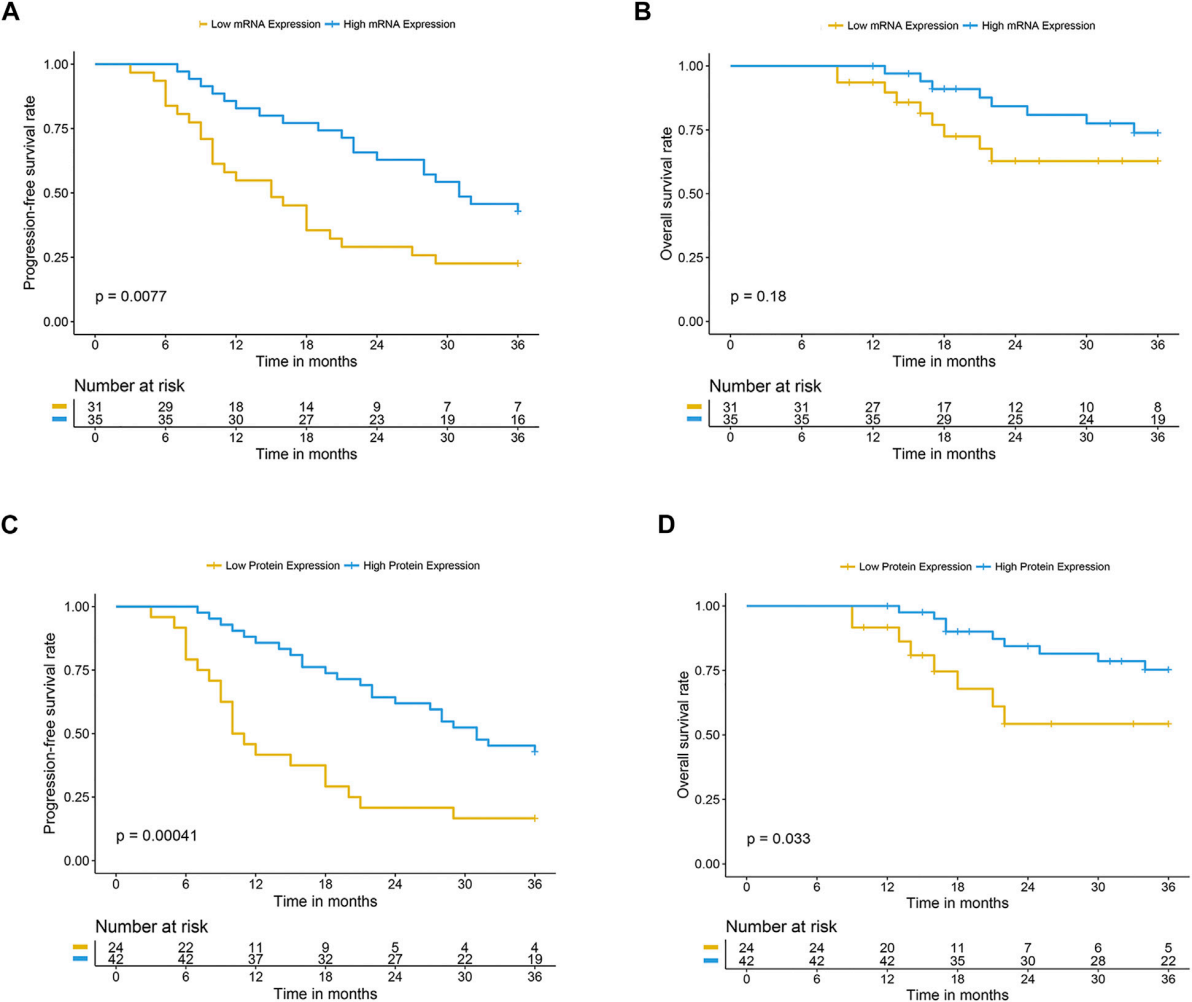
Clinical outcome of patients with high and low expression of OCTN2 treated with oxaliplatin. Kaplan -Meier progression-free survival and overall survival curves in OCTN2 low- and high-expressing groups defined according to the relative mRNA level **(A** and **B)** or the staining score **(C** and **D)**. Samples with relative mRNA value or staining score ≥ of the median value were classified as OCTN2 high-expressing tumors.

**FIGURE 5:**
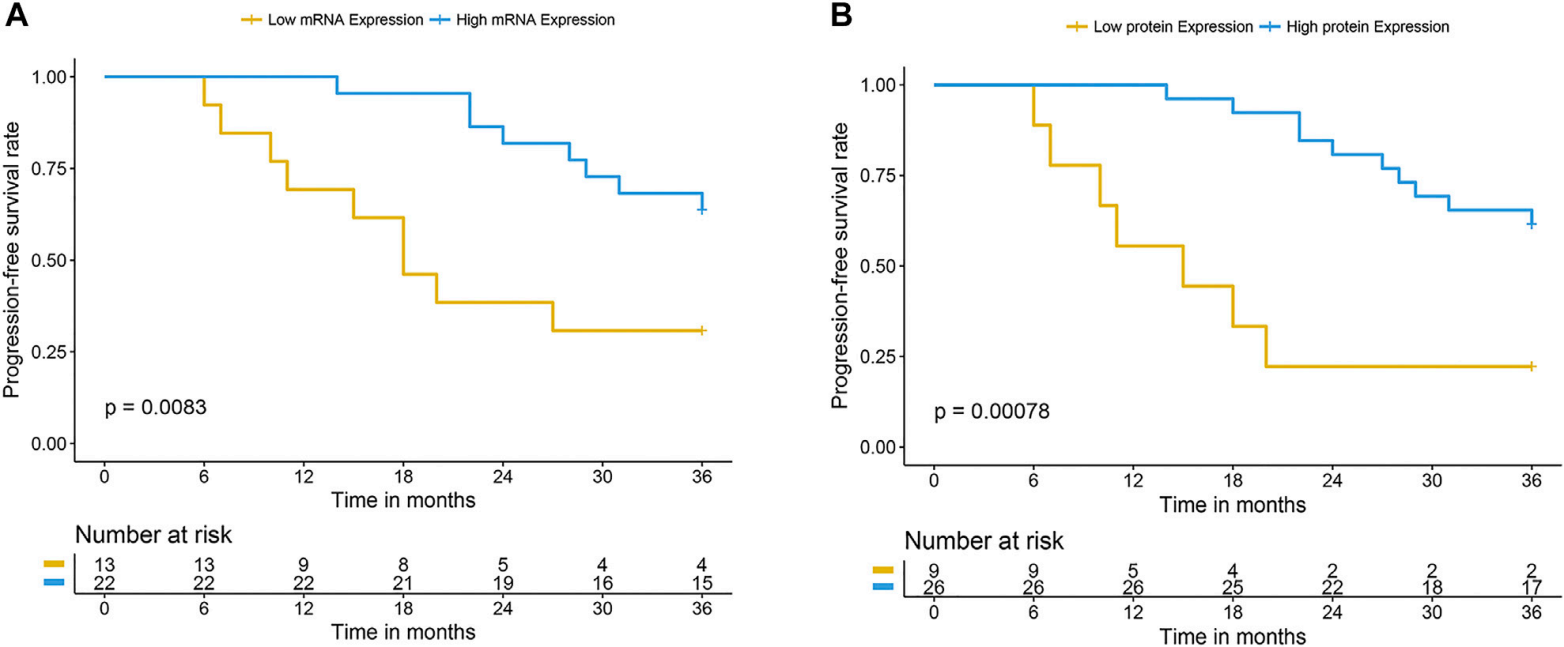
PFS of patients with high and low expression of OCTN2 with negative lymph nodes treated with oxaliplatin. Kaplan -Meier progression-free survival curves in lymph node negative patients with low or high mRNA **(A)** or protein **(B)** expression of OCTN2. Samples with relative mRNA value or staining score ≥ of the median value were classified as OCTN2 high-expressing tumors.

The differential expression level of OCTN2 in cancerous and normal mucosa was confirmed in additional 67 SCC and 22 AC samples that were retrospectively analyzed (Figure 3). For the 67 SCC samples a 3-year clinical follow-up of the patients was available. The follow-up period for all 67 patients was 9–36 months. Recurrence or distant metastases occurred in 45 patients (67%), and 40 (60%) had died by the end of the follow-up period. One patient was excluded from the survival analysis due to the presence of metastasis at the time of the diagnosis. Progression-free survival (PFS) at 1 and 2 years was 70 and 44%, respectively, with a median PFS of 25 months. Overall survival (OS) at 1 and years was 89 and 56%, respectively, with a median OS of 27 months. Among the clinicopathologic factors included in the analysis, the lymph node status, and tumor location were found the most relevant parameter affecting the clinical outcome. Patients with positive lymph node(s) had a significantly higher risk of recurrence (HR = 3.32, 95% CI 1.79–6.15) in comparison with those with negative lymph node(s). Patients with mid-thoracic tumor had a longer overall survival than those with upper-thoracic location (HR = 0.23, 95% CI 0.06–0.91) (Table 2). The mRNA and protein levels of OCTN2 were independent from the lymph node status and the tumor location (Supplementary Table S1; Supplementary Figure S2) yet associated with the clinical outcome (Table 2; Supplementary Table S2). Patients with OCTN2 high-expressing tumor had a significantly lower risk of recurrence (HR = 0.35, 95% CI 0.19–0.64) and death (HR = 0.37, 95% CI 0.14–0.96) (Table 2; Figure 4). Both associations held up in multivariate analysis, indicating that lymph node status and OCTN2 expression level were independent risk factors of recurrence and death (Table 3; Supplementary Table S3). Notably, OCTN2 expression level did affect the PFS of patients with negative lymph node(s) but not that of patients with infiltrated lymph node(s) (Figure 5).

**TABLE 2:** Univariate analysis hazard ratios from the Cox PH model.

	Progression-free survival (*n* = 66)			Overall survival (*n* = 66)		
Covariate	HR	95% CI	*p*-value	HR	95% CI	*p*-value
Age	1.46	0.81–2.64	0.21	2.68	0.99–7.26	0.05
Location						
Upper	−	−	−	−	−	−
Medium	0.67	0.33–1.35	0.26	0.23	0.06–0.91	**0.04**
Lower	0.88	0.41–1.90	0.74	1.08	0.38–3.08	0.89
Invasion						
T1-T2	−	−	−	−	−	−
T3-T4	1.58	0.78–3.20	0.20	3.64	0.83–15.94	0.09
Node						
N0	−	−	−	−	−	−
N1-N3	3.32	1.79–6.15	**0.0001**	2.95	2.15–8.31	**0.03**
Grade						
G1-G2	−	−	−	−	−	−
G3-G4	0.91	0.49–1.69	0.76	0.23	0.05–0.99	0.05
OCTN2 staining						
Low	−	−	−	−	−	−
High	0.35	0.19–0.64	**0.0006**	0.37	0.14–0.96	**0.04**

HR, Hazard ratio; CI, Confidence interval. *p*-values < 0.05 are highlighted in bold.

**TABLE 3:** Multivariate analysis hazard ratios from the Cox PH model.

	Progression-free survival (*n* = 66)			Overall survival (*n* = 66)		
Covariate	HR	95% CI	*p*-value	HR	95% CI	*p*-value
Location						
Upper	−	−	−	−	−	−
Medium	0.44	0.21–0.93	**0.03**	0.15	0.04–0.61	**0.009**
Lower	0.74	0.34–1.61	0.45	0.96	0.36–2.94	0.96
Node						
N0	−	−	−	−	−	−
N1-N3	2.81	1.34–5.07	**0.005**	1.89	0.64–5.56	0.25
OCTN2 staining						
Low	−	−	−	−	−	−
High	0.37	0.18–0.74	**0.005**	0.24	0.08–0.74	**0.013**

*p*-values < 0.05 are highlighted in bold.

## Discussion

This study demonstrates that the Carnitine/Organic Cation Transporter Novel 2 (OCTN2) is expressed and functioning in esophageal tumors. Notably, patients with SCC whose tumor cells are characterized by high expression level of OCTN2 have a better response to oxaliplatin-based chemotherapy, with a significant prolongation of PFS and OS. OCTN2 has been previously shown to transport oxaliplatin in HEK-OCTN2 cells (Jong et al., 2011), therefore it is conceivable that in tumors with higher expression of OCTN2, higher intracellular level of oxaliplatin can be reached, resulting in a stronger cytotoxic effect. Indeed, both primary patient-derived cancer cells with higher OCTN2 expression and activity, as well as HEK293 stably transfected with OCTN2, are markedly more sensitive to oxaliplatin. Our data further advocate the critical role of OCTs in cancer chemotherapy sensitivity/resistance. It has been recently shown that OCTN1 can facilitate the intracellular accumulation of the pyrimidine nucleoside analog cytarabine, and that higher expression of OCTN1 is associated with a longer survival in patients with acute myeloid leukemia treated with this drug (Drenberg et al., 2017). Analogously, epigenetic activation of OCT2, which is often repressed in renal cell carcinoma (Winter et al., 2016; Visentin et al., 2018) sensitizes renal carcinoma cells to oxaliplatin, suggesting that OCT2 expression level is critical in chemosensitivity in renal cell carcinoma (Liu et al., 2016). OCTN2 expression level has been recently shown to be higher in more aggressive glioblastomas, hence representing a negative prognostic marker in these patients, likely because of a higher accumulation of L-carnitine, which is an important co-factor in energy metabolism and a potent antioxidant (Gulcin, 2006; Fink et al., 2019). In fact, OCTN2 transports with high-affinity carnitine and its precursor γ-butyrobetaine (Tamai et al., 1998; Fujita et al., 2009). Two major genetic evidences underpin the pivotal role of OCTN2 in carnitine homeostasis in eukaryotes: (i) children diagnosed with systemic carnitine deficiency (OMIM212149) carry loss-of-function mutations in the SLC22A5 gene (Nezu et al., 1999), (ii) the juvenile visceral steatosis (jvs) mouse, characterized by impaired intestinal absorption, tissue distribution and reabsorption of carnitine, lack the octn2 transporter (Koizumi et al., 1988; Horiuchi et al., 1993; Hashimoto et al., 1998; Yokogawa et al., 1999; Tamai et al., 2001; Kato et al., 2006). Even though OCTN2 expression level does not seem to affect the proliferation of primary patient-derived cancer cells, it cannot be excluded esophageal cancers with high expression of OCTN2 and high accumulation of L-carnitine being less aggressive than those expressing low levels of the transporter, which would make OCTN2 also a candidate prognostic marker. To understand the impact of OCTN2 on tumor biology, the impact of OCTN2 expression on the clinical outcome of oxaliplatin-naïve patients with esophageal cancer must be investigated.

The overexpression of OCTN2 in patients with esophageal cancer has also important ramifications in diagnostic imaging and pathology. The standard methods for staging esophageal cancer are endoscopic ultrasonography and computed tomography (Jayaprakasam et al., 2020). Both techniques entirely depend on physical characteristics of the lesion, resulting in diagnostic performance limitations. Metabolic assessment of esophageal cancers by positron emission tomography (PET) with ^18^F-2-Fluorodeoxyglucose showed some value, especially in the detection of long distance metastasis (Flamen et al., 2000). Because cellular uptake is often rate-limiting in tracer accumulation (Mochizuki et al., 2001; Song et al., 2008; Hiyoshi et al., 2009; Kaira et al., 2010; Pastor et al., 2014; Visentin et al., 2017b), differential expression in key membrane transporters can be exploited to develop rationally designed imaging probes. Assessing L-carnitine accumulation by PET might not only improve the sensitivity in the visualization of long distance metastasis, but also guide the oncologist in the selection of those patients who are more likely to respond to oxaliplatin-based chemotherapy by providing a live assessment of the function of OCTN2, an information that cannot be obtained by standard diagnostic pathology. Noteworthy, such approach requires the fluorination of L-carnitine to achieve a longer half-life of the radioisotope (2 h) in comparison with that of the carbon-11-labeled (20 min). The experimental assessment of the impact of such chemical modification on the affinity for OCTN2 is a prerequisite for future clinical application (Visentin et al., 2017b; Visentin et al., 2018).

OCTN2-mediated transport is inhibited by several clinically used drugs such as valproic acid, amisulpride, mildronate, the β-lactam, and polymyxin antibiotics, and the anticancer drugs vinca alkaloids, taxanes, daunorubicin (Tamai, 2013; Visentin et al., 2017a). Our findings indicate that the co-administration of any of the above-mentioned drugs during the treatment with oxaliplatin might jeopardize the therapeutic outcome. Conversely, the DNA topoisomerase II inhibitor etoposide, which is a substrate of OCTN2 and showed some encouraging results in the treatment of esophageal cancer, might be part of novel rationally designed therapeutic regimens to add to the current treatment arsenal against esophageal carcinoma (Kok et al., 1988; Ohwada et al., 1995; Kok et al., 1996; Hu et al., 2012; Tamai, 2013; Yamamoto et al., 2018). It is also intriguing to hypothesize that the pharmacological induction of OCTN2 might sensitize the cancer cells to oxaliplatin. While the transcriptional regulation of OCTN2 in esophageal cancer cells has not been studied yet, candidate pathways can be inferred from previous studies in other tissues and diseases. In normal tissues the transcription of SLC22A5, the gene encoding for OCTN2, is primarily governed by the peroxisome proliferator-activated receptor family. In the liver and in the small intestine OCTN2 transcriptional regulation is under control of the peroxisome proliferator-activated receptor-α (PPARα) (Ringseis et al., 2007; Koch et al., 2008). In the colon instead, PPARγ seems to be the main determinant of the transcriptional regulation of OCTN2 (D'Argenio et al., 2010). Consistently, pharmacological activation of PPARγ increased mRNA and protein expression of OCTN2, and oxaliplatin sensitivity in colorectal cancer SW480 cells (Qu et al., 2014).

This study has limitations, such as the lack of information on the impact of OCTN2 on the clinical outcome of patients with esophageal adenocarcinoma treated with oxaliplatin as well as the lack of oxaliplatin-naïve groups important to better understand the impact of OCTN2 on tumor biology. Nonetheless, our results completes the previous observation that esophageal cancers expressing the P-type ATPase ATP7B, a platinum exporter, are more resistant to cisplatin than ATP7B-negative tumors (Higashimoto et al., 2003; Burger et al., 2011). Taken together, these results lay the foundation for future confirmatory prospective studies whose results can change the overall approach to this disease from the diagnosis to the follow-up, striving for more accurate staging and re-staging, optimal selection of responders, and non-responders and improved detection of recurrence.

## Data Availability

The raw data supporting the conclusions of this article will be made available by the authors, without undue reservation.
