# Supplementary material for: The Role of the Carnitine/Organic Cation Transporter Novel 2 in the Clinical Outcome of Patients With Locally Advanced Esophageal Carcinoma Treated With Oxaliplatin
Source: Front Pharmacol. 2021 Sep 16;12:684545. doi: 10.3389/fphar.2021.684545 (PMC8481660; doi:10.3389/fphar.2021.684545)
Supplement: Supplementary file 1 [file Table1.docx]

**Table S1. Summary of chi-square test for independence.**

|  | OCTN2 protein level (n=109) | | | OCTN2 mRNA level (n=67) | | |
| --- | --- | --- | --- | --- | --- | --- |
| Variable | **χ^2^** | **df** | ***P*-value** | **χ^2^** | **df** | ***P*-value** |
| Age | 0.48 | 1 | 0.49 | 0.62 | 1 | 0.43 |
| Location | 2.12 | 2 | 0.35 | 2.36 | 2 | 0.31 |
| Invasion (T) | 0.24 | 1 | 0.63 | 0.001 | 1 | 0.98 |
| Node (N) | 0.80 | 1 | 0.37 | 2.11 | 1 | 0.15 |
| Grade | 0.41 | 1 | 0.52 | 0.13 | 1 | 0.72 |

df, degree of freedom
