# Supplementary material for: The Role of the Carnitine/Organic Cation Transporter Novel 2 in the Clinical Outcome of Patients With Locally Advanced Esophageal Carcinoma Treated With Oxaliplatin
Source: Front Pharmacol. 2021 Sep 16;12:684545. doi: 10.3389/fphar.2021.684545 (PMC8481660; doi:10.3389/fphar.2021.684545)
Supplement: Supplementary file 2 [file Image2.pdf]

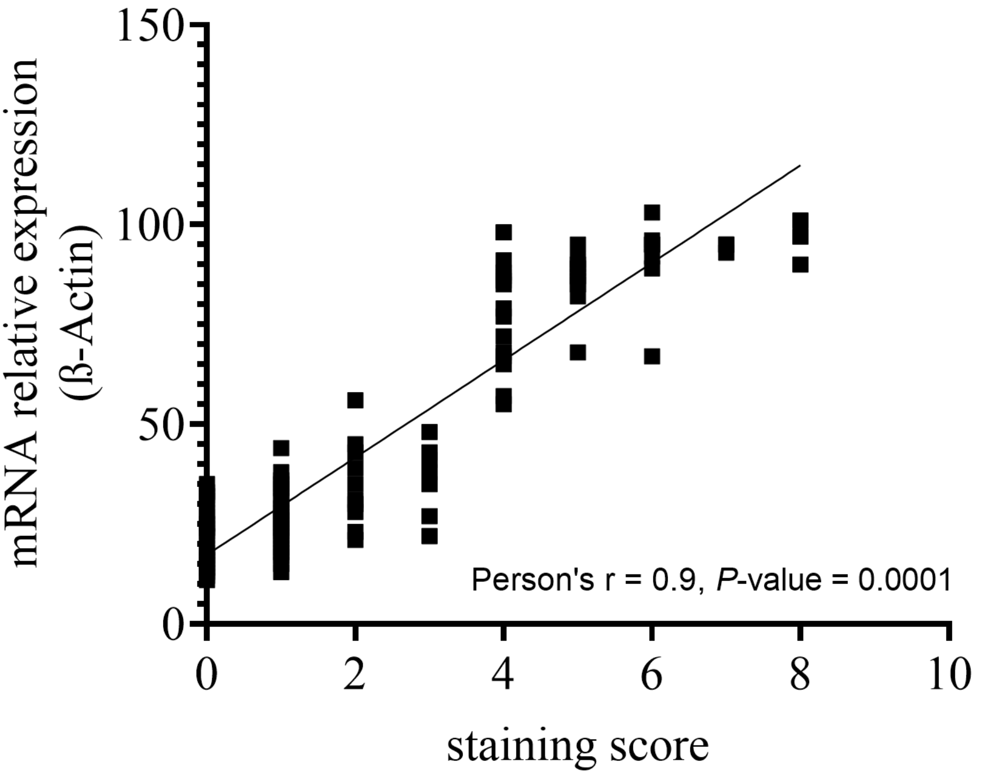


**Figure S2. Correlation between OCTN2 protein and mRNA level.** mRNA and protein level of 67 SCC samples and the matched normal tissues were correlated by Pearson's Rank - Order analysis.
