# Supplementary material for: The Role of the Carnitine/Organic Cation Transporter Novel 2 in the Clinical Outcome of Patients With Locally Advanced Esophageal Carcinoma Treated With Oxaliplatin
Source: Front Pharmacol. 2021 Sep 16;12:684545. doi: 10.3389/fphar.2021.684545 (PMC8481660; doi:10.3389/fphar.2021.684545)
Supplement: Supplementary file 3 [file Table2.docx]

**Table S2. Univariate survival analysis relative to OCTN2 mRNA level.**

|  | Progression-Free Survival (n=66) | | |  | Overall Survival (n=66) | | |
| --- | --- | --- | --- | --- | --- | --- | --- |
| Covariate | **HR** | **95% CI** | ***P*-value** |  | **HR** | **95% CI** | ***P*-value** |
| OCTN2 mRNA  Low  High | -  0.45 | **-**  0.25-0.82 | **0.009** |  | 0.52 | 0.20-1.36 | **0.18** |

HR, Hazard ratio; CI, Confidence interval
