# Supplementary material for: The Role of the Carnitine/Organic Cation Transporter Novel 2 in the Clinical Outcome of Patients With Locally Advanced Esophageal Carcinoma Treated With Oxaliplatin
Source: Front Pharmacol. 2021 Sep 16;12:684545. doi: 10.3389/fphar.2021.684545 (PMC8481660; doi:10.3389/fphar.2021.684545)
Supplement: Supplementary file 4 [file Table3.docx]

**Table S3. Multivariate survival analysis relative to OCTN2 mRNA level.**

|  | Progression-Free Survival (n=66) | | |  | Overall Survival (n=66) | | |
| --- | --- | --- | --- | --- | --- | --- | --- |
| Covariate | **HR** | **95% CI** | ***P*-value** |  | **HR** | **95% CI** | ***P*-value** |
| Location  Upper  Medium  Lower | -  0.50  0.81 | -  0.24-1.03  0.37-1.78 | -  0.06  0.60 |  | -  0.18  1.10 | -  0.05-0.72  0.38-3.23 | -  **0.016**  0.86 |
| Node  N0  N1-N3 | -  2.92 | -  1.54-5.57 | **^-^**  **0.001** |  | -  2.54 | -  0.93-6.94 | **-**  **0.07** |
| OCTN2 mRNA  Low  High | -  0.49 | **-**  0.25-0.95 | **-**  **0.036** |  | -  0.39 | -  0.14--1.10 | **-**  **0.07** |

HR, Hazard ratio; CI, Confidence interval
