# Supplementary material for: The Role of the Carnitine/Organic Cation Transporter Novel 2 in the Clinical Outcome of Patients With Locally Advanced Esophageal Carcinoma Treated With Oxaliplatin
Source: Front Pharmacol. 2021 Sep 16;12:684545. doi: 10.3389/fphar.2021.684545 (PMC8481660; doi:10.3389/fphar.2021.684545)
Supplement: Supplementary file 5 [file Image1.pdf]

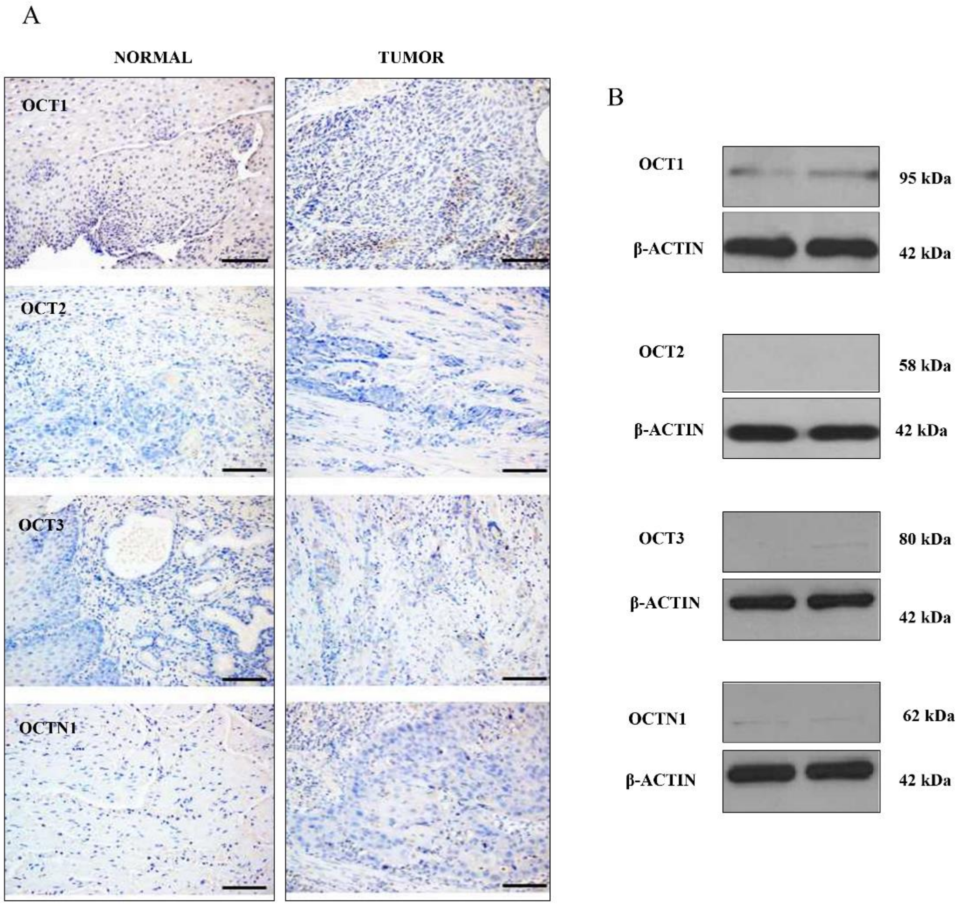

Figure S1. Protein expression level of organic cation transporters in SCC samples. Representative images of the immunostaining for OCT1, OCT2, OCT3 and OCTN1 in matched adjacent normal tissues and tumor tissues from patients with SCC who underwent partial esophagectomy. Scale bar=100 μm (A). Representative western blot of OCT1, OCT2, OCT3 and OCTN1 from matched adjacent normal tissue and tumor tissue of patients with SCC (B).
